# The risk factors of cardiovascular disease in patients with renal transplantation

**DOI:** 10.12669/pjms.306.5610

**Published:** 2014

**Authors:** Hongming Zhang, Xiaoyan Li

**Affiliations:** 1 Hongming Zhang, Department of Cardiology, The General Hospital of Jinan Military Region. Jinan, Shandong, 250031, P.R. China.; 2 Xiaoyan Li, Department of Cardiology, The General Hospital of Jinan Military Region. Jinan, Shandong, 250031, P.R. China.

**Keywords:** Coronary heart disease, Kidney transplantation, Risk factors

## Abstract

***Objective*** *:* This study aims to observe the risk factors of cardiovascular disease in patients after kidney transplantation.

***Methods:*** Total 102 patients after renal transplantation (group A) and 96 clinic examination cases (group B) were recruited. Blood pressure, blood urea nitrogen (BUN) and creatinine (Cr), blood glucose (GLU), total cholesterol (TC), triglyceride (TG), high density lipoprotein cholesterol (HDL-C), low density lipoprotein cholesterol (LDL-C) and apolipoprotein A-I ( apoA-I ) of the subjects were tested. These indexes were tested 3 times in group A in the 1, 2, and 3 month after kidney transplantation surgery. Electrocardiogram examination was done in the third month after kidney transplantation surgery.

***Results:*** There were significant differences in the incidence of hypertension, hyperglycemia and dyslipidemia between the two groups. After operation, the levels of BUN and Cr decreased significantly in 57 cases of group A without taking antihypertensive drugs (*P*<0.01). However, there was no significant difference in the blood pressure regardless of the systolic blood pressure (SBP) and diastolic blood pressure (DBP) before and after operation. In group A, the abnormal rate of ECG was 65%, the incidence of ST-T changes, prolongation of QT interval, left ventricular hypertrophy and left ventricular high voltage increased significantly (*P*<0.01).

***Conclusion:*** The incidence of coronary heart disease risk factors such as hypertension and hyperglycemia are high in patients after renal transplantation.

## INTRODUCTION

The morbidity of cardiovascular complications is very high in patients with chronic renal failure and uremia. The 5 years survival rate of uremia patients has increased in recent years along with the improving dialysis technology, but it does not reduce the cardiovascular complications. Cardiovascular disease is still a major cause of morbidity and mortality in patients with end stage renal disease^1^^-^^3^, and it is the leading cause of death following renal transplantation accounting for 40% to 55% of all deaths.^4^^,^^5^ Hypertension, hyperglycemia and dyslipidemia are widely accepted coronary heart disease risk factors. These risk factors accumulated in patients after renal transplantation. This study aims to observe the traditional cardiovascular risk factors among renal transplant recipients, which can provide clues for treatments of patients with end stage renal disease to increase their survival rate.

## METHODS

Data for this study was obtained from the general hospital of Jinan military region. The ethics committee of the General Hospital of Jinan Military Command approved the study protocols and all patients gave informed consent. A total of 102 patients (78 male and 24 female; mean age = 40 ± 4.9 years) diagnosed with chronic glomerular nephritis uremia who underwent renal transplantation, as well as 96 healthy controls (54 male and 42 female; mean age = 37.9 ±7.2 years) were recruited from July 2010 to September 2012. The patients were on regular dialysis before renal transplantation with 2 ~ 3 times a week. Their durations of illness were 38 months. There was no significant difference between two groups in gender, age and body weight. 

The daily morning resting blood pressure, blood urea nitrogen (BUN) and creatinine (Cr) of all patients were measured one week before the operation and for 3 consecutive times. Their average values were the preoperative mean preoperative blood pressure, BUN and Cr respectively. At the last week of 1^st^ 2^nd^ and 3^rd^ month after transplantation, BUN and Cr of all patients were measured two times and their average values were the BUN and Cr values of that month, blood pressure were measured at least 3 times daily and their average values were the blood pressure values of that month. 12 lead ECG was done for all the patients and three 3 months. Glucose (GLU), total cholesterol (TC), three triacylglycerol (TG), high density lipoprotein cholesterol (HDL-C), low density lipoprotein cholesterol (LDL-C) and apolipoprotein A - I (apoA- I) were collected.

Transplant operation mode was conventional right abdominal incision, cadaveric kidney were used, donor renal vein and external iliac vein were end-to-side anastomosis, donor renal artery and internal iliac artery were also end to side anastomosis. Pathogenic kidney was not resected.

In 102 cases of renal transplantation, 57 patients had no antihypertensive therapy in 10 days before operation and the observation period of after operation. Diagnostic criteria of hypertension used was the new diagnostic criteria of WHO and the international society of hypertension. Glucose was determined with venous plasma. It was thought to be high when it was greater than 5.6 mmol / L.

***Statistical Analysis:*** Differences in patient characteristics and clinical variables were analysed using the 2 test and ANOVA. Data analyses were performed with commercially available statistical software (11.0, SPSS, Chicago, IL, USA).

## RESULTS

The morbidity of high blood glucose and blood lipid abnormality in patients after renal transplantation was significantly higher than that of the control group (Table-I). A total of 95 patients after renal transplantation were diagnosed as hypertensives according to the diagnostic criteria of hypertension. The BUN and Cr in the 57 cases of 102 renal transplantation cases who were not treated with any antihypertensive therapy in 10 days before operation and the observation period of after operation significantly decreased after operation comparing with that before operation. However, there were no differences in their systolic and diastolic blood pressure before and after operation (Table-II).

The abnormal rate of electrocardiogram (ECG) in group A was 65%. The incidence of ST-T changes, prolongation of the QT interval, left ventricular hypertrophy and with high voltage was higher in group A than that in control group significantly (*P*<0.01).

## DISCUSSION

Renal transplantation is one of the most effective treatments for most patients with end-stage renal disease. The survival rate of patients undergoing renal transplantation has improved considerably over the past decades. Patients with end-stage renal disease often suffer from complications with cardiovascular disease, which is much more common in transplant patients. Its contribution to post-transplant mortality to some extent reflects the incidence of cardiac disease in the general population.^5^^,^^6^ Our investigation found that the morbidity of hypertension, hyperglycemia and dyslipidemia among renal transplant recipients was very high, which confirmed that the risk factors of cardiovascular disease still exist in transplant patients.

**Table-I:** Comparison of the risk factors of cardiovascular disease in two groups

**Group**	**Cases**	**High GLU cases (%)**	**Low HDL-C cases (%)**	**High TC cases (%)**	**High TG cases (%)**	**High LDL-C cases (%)**	**Low ** **apoA-I** ** cases (%)**
A	102	12(11.76) *	11(10.78)*	16(15.69) *	13(12.74) *	9(8.82) *	10(9.80) *
B	96	3(3.12)	4(4.17)	3(3.12)	3(3.12)	2(2.08)	3(3.12)

group A: patients after renal transplantation, group B: Control.

GLU: blood glucose, HDL-C: high density lipoprotein cholesterol, TC: total cholesterol, TG: triglyceride, LDL-C: low density lipoprotein cholesterol, apoA-I: apolipoprotein A-I.

* P<0.05

**Table-II:** Comparison of the blood pressure and renal function before and after renal transplantation in 57 cases not treated with any antihypertensive therapy in 10 days before operation and the observation period of after operation (Mean ± SD).

**Before renal transplantation**		**After renal transplantation**		
		**1 month**	**2 month**	**3 month**
BUN(mmol/L)	26.44±6.76	7.42±4.07**	6.97±6.23**	7.04±3.52**
Cr(umol/L)	971.78±157.77	135.56±52.99**	123.56±47.65**	132.31±44.35**
SDP(mmHg)	149.20±17.06	154.60±18.48	152.31±17.87	146.82±20.03
DBP(mmHg)	97.57±11.46	102.27±11.40	97.09±12.82	96.67±12.71

BUN: blood urea nitrogen, Cr: creatinine, SDP: Systolic blood pressure, DBP: diastolic blood pressure

** P<0.01

The incidence of hypertension in patients after renal transplantation was more than 40%.^7^^,^^8^ We observed that the incidence of hypertension was as high as 93% after renal transplantation for 3 months. There was no reduction in blood pressure although their BUN and Cr decreased and urine volume increased significantly. Hypertension in the renal transplant patients represents a greater risk for cardiovascular outcome and renal survival than it does in the common population and it plays a major role in the causation of chronic graft dysfunction, morbidity and deaths from cardiovascular disease.^9^ Many causes for post transplant hypertension such as the interaction between recipient and donor, hypercalcemia and recurrence of glomerular nephritis are well-documented in the literature.^8^^-^^11^ Persistent hypertension after renal transplantation has serious harm to the function of heart, brain and kidney function of transplant patient. We found that the abnormal ECG rate was as high as 65% in transplant patients, which was related with persistent hypertension undoubtedly. 

Previous studies have found that renal transplantation patients had complications of hyperlipidemia,^12^ had a high prevalence of glucose metabolism disorders^13^ and had atherosclerosis.^14^ We found that the incidence of hyperlipidemia especially high cholesterol and high triglycerides was significantly higher in patients after renal transplantation than that in the general population. Our data also showed that the morbidity of hyperglycemia in the renal transplantation patients was higher than that of control group. These may promote the occurrence and development of coronary heart disease. 

In summary, we observed the traditional cardiovascular risk factors among renal transplant recipients in this study. We found that the incidence of cardiovascular disease continues to be high after renal transplantation. Therefore, treatment of risk factors must be effective and introduced early in the course of renal failure and continued following transplant to increase their survival rate.

## References

[1] Kasiske BL, Guijarro C, Massy ZA, Wiederkehr MR, Ma JZ (1996). Cardiovascular disease after renal transplantation. J Am Soc Nephrol.

[2] Mazzarella V, Danese V, Pisani F, Buonomo O, Pollicita S, Rodio F (2001). Echographic valuation of risk factors for cardiovascular disease in patients with renal transplantation. Transplant Proc.

[3] Lemieux I, Houde I, Pascot A, Lachance JG, Noël R, Radeau T (2002). Effects of prednisone withdrawal on the new metabolic triad in cyclosporine-treated kidney transplant patients. Kidney Int.

[4] Watorek E, Szymczak M, Boratynska M, Patrzalek D, Klinger M (2011). Cardiovascular risk in kidney transplant recipients receiving mammalian target of rapamycin inhibitors. Transplant Proc.

[5] Ostovan MA, Fazelzadeh A, Mehdizadeh AR, Razmkon A, Malek-Hosseini SA (2006). How to decrease cardiovascular mortality in renal transplant recipients. Transplant Proc.

[6] Fox H, Büttner S, Hemmann K, Asbe-Vollkopf A, Doss M, Beiras-Fernandez A (2013). Transcatheter aortic valve implantation improves outcome compared to open-heart surgery in kidney transplant recipients requiring aortic valve replacement. J Cardiol.

[7] Castillo-Lugo JA, Vergne-Marini P (2005). Hypertension in kidney transplantation. Semin Nephrol.

[8] Kasiske BL, Anjum S, Shah R, Skogen J, Kandaswamy C, Danielson B (2004). Hypertension after kidney transplantation. Am J Kidney Dis.

[9] Barbari A (2013). Posttransplant hypertension: multipathogenic disease process. Exp Clin Transplant.

[10] Morath C, Schmied B, Mehrabi A, Weitz J, Schmidt J, Werner J (2009). Angiotensin-converting enzyme inhibitors and angiotensin II type 1 receptor blockers after renal transplantation. Clin Transplant.

[11] Ferro CJ, Savage T, Pinder SJ, Tomson CR (2002). Central aortic pressure augmentation in stable renal transplant recipients. Kidney Int.

[12] Trespalacios FC1, Taylor AJ, Agodoa LY, Abbott KC (2002). Incident acute coronary syndromes in chronic dialysis patients in the United States. Kidney Int.

[13] Brzezińska B, Junik R, Kamińska A, Włodarczyk Z, Adamowicz A (2013). Factors associated with glucose metabolism disorders after kidney transplantation. Endokrynol Pol.

[14] Turkmen K, Erdur FM, Guney I, Ozbiner H, Toker A, Gaipov A (2012). Relationship between Plasma Pentraxin-3, Neutrophil-to-Lymphocyte Ratio, and Atherosclerosis in Renal Transplant Patients. Cardiorenal Med.

